# Genome-Wide Identification and Expression Analysis of *MRLK* Family Genes Associated with Strawberry (*Fragaria vesca*) Fruit Ripening and Abiotic Stress Responses

**DOI:** 10.1371/journal.pone.0163647

**Published:** 2016-09-29

**Authors:** Qing Zhang, Meiru Jia, Yu Xing, Ling Qin, Bingbing Li, Wensuo Jia

**Affiliations:** 1 College of Horticulture, China Agriculture University, Beijing, China; 2 College of Plant Science and Technology, Beijing University of Agriculture, Key Laboratory of New Technology in Agricultural Application of Beijing, Beijing University of Agriculture, Beijing, China; 3 Beijing Collaborative innovation center for eco-environmental improvement with forestry and fruit trees, Beijing, China; Institute of Genetics and Developmental Biology Chinese Academy of Sciences, CHINA

## Abstract

Malectin-like domain-containing receptor-like kinases (MRLK) constitute a large and divergent family of proteins in plants; however, little is known about the role of MRLKs in fruit growth and development. In this study, we characterized *MRLK* family genes in diploid strawberry, *Fragaria vesca*. Based on an analysis of malectin-like domain and a search in the strawberry genome and NCBI database, we identified 62 *FvMRLKs* in the strawberry genome, and classified these genes into six subfamilies with distinct malectin domains in the extracellular regions of the encoded proteins. Gene expression analysis indicated that more than 80% of the *FvMRLKs* were expressed in various tissues, with higher levels in roots than in other organs. Thirty-three *FvMRLKs* were found to be expressed in fruits during the early stages of development, and over 60% of these exhibited dramatic decreases in expression during fruit growth and development. Moreover, the expression of some *FvMRLKs* was sensitive to both environmental and internal cues that play critical roles in regulating strawberry fruit development and ripening. Collectively, this study provides valuable insight into the *FvMRLKs* gene family and its role in regulating strawberry fruit development and ripening.

## Introduction

Receptor-like kinases (RLKs) in plants comprise a large family of proteins with putative amino-terminal extracellular domains and carboxyl-terminal intracellular kinase domains [1, 2]. A phylogenetic analysis of the model plant *Arabidopsis thaliana* identified more than 610 proteins containing RLK domain sequences. RLKs can be further divided into more than 44 subfamilies based on their extracellular domain structures [3, 4]; plant RLKs were initially categorized into three major types, the S-domain type, the leucine-rich repeat type, and the epidermal growth factor (EGF)-like type [5, 6]. CrRLK1 was later identified in *Catharanthus roseus* and represents a unique subfamily of RLKs [5].

The *Arabidopsis* genome contains 17 *CrRLK1*-like *RLK* genes. The *CrRLK1L* subfamily can be further classified into six subclasses [7, 8].The functions of several members of the *CrRLK1L* subfamily have been determined in *Arabidopsis* [7]. *Feronia* (FER) was first identified for its role in fertilization [9]. Recent research demonstrated that FER directly interacts with guanine nucleotide exchange factors (ROPGEFs), which regulate RHO GTPase signaling and mediate the production of reactive oxygen species (ROS) at the entrance point of the female gametophyte, which induces pollen tube rupture and sperm release [10–12]. Besides its critical roles in fertilization, FER has been suggested to be a key regulator of the crosstalk among abscisic acid (ABA), brassinosteroids (BR), and ethylene signaling [13]. Moreover, FER was reported to be involved in a variety of important processes, such as root hair elongation[14,15], ethylene biosynthesis [16], starch accumulation[17], seed development [18], pathogen resistance[19, 20], and vegetative growth [21–23].Theseus 1 (THE1) was identified in a screen for suppressors that attenuated the short hypocotyl phenotype of dark-grown seedlings [24–26].THE1 functions as a cell wall integrity sensor that mediates the disruption of cellulose synthesis [24–26].Named after the male consort of Feronia, Anxur1 (ANX1) and Anxur2 (ANX2) are closely related to FER. Like FER, ANX1 and ANX2 are pollen-specific CrRLK1Ls that play important roles in the regulation of pollen tube rupture and sperm release [27]. HERCULES 1 (HERK1) is also a member of the CrRLK1L subfamily and functions redundantly with THE1 to regulate plant growth and development [21, 22].

Acting as RLKs, CrRLK1Ls signaling is based on a reaction with specific ligands in the extracellular region of their conserved domains; therefore, analysis of the conserved domains of CrRLK1Ls is particularly important for a deeper understanding of the mechanisms underlying CrRLK1L signaling. Sequence analysis of FER, THE1, ANXS, HERK1, and several other closely related proteins indicated that an important feature of their extracellular sequences is the existence of a conserved domain, named the malectin or malectin-like domain. Malectin is a membrane-anchored protein of the endoplasmic reticulum. It recognizes and binds Glc2-N-glycan, thereby regulating the production and secretion of *N*-glycosylated proteins [28–30]. The existence of the malectin domain provides an important clue for exploring the signaling mechanisms of RLKs. CrRLK1Ls are commonly referred to as malectin domain-containing RLKs [7, 25, 31]. Given the importance of CrRLK1Ls, genome-wide identification and analysis of the *CrRLK1L*gene subfamily has been conducted in several plant species, including *Oryza sativa* (rice) [32] and *Gossypium* (cotton) [33]. As mentioned above, the *Arabidopsis* genome contains 17 *CrRLK1*-like *RLK* genes. However, a search for malectin and malectin-like domains in *Arabidopsis* revealed more than 30 genes, indicating that malectin and malectin-like domain containing RLKs exist outside of the *CrRLK1L*gene family. Therefore, it is more suitable to categorize the malectin and malectin-like domain containing RLKs into a unique subfamily (referred as *MRLK* subfamily) and a genome-wide analysis of this gene subfamily would provide insight into their functions in plants.

Flesh fruit production accounts for a substantial fraction of the world’s agricultural output. Since regulating fruit development and ripening is a key challenge in fleshy fruit production, understanding the molecular basis for fruit development and ripening is of great interest. While signaling mechanisms have been extensively studied for the regulation of plant growth and development and responses to abiotic and biotic stresses, limited information is available in fleshy fruits, especially non-climacteric fruits. Fleshy fruits are physiologically classified as climacteric or non-climacteric. Strawberry is becoming a model plant for basic research of non-climacteric fruits (i.e., fruits that ripen without increased ethylene production).To explore the molecular basis of strawberry plant growth and development, with a focus on fruit growth and development, we conducted a genome-wide analysis of the *MRLK* subfamily in diploid strawberry, *Fragaria vesca*. We found that the *F*. *vesca* genome contains more than 60 *MRLKs*, about 50% of which are expressed in the fruit. Among the *MRLKs* expressed in fruit, more than 60% were expressed at relatively high levels only in the early stages of fruit development, with transcript levels declining dramatically as fruit development progressed. Furthermore, many of these genes were sensitive to both environmental and internal cues controlling fruit development and ripening. These findings provide valuable insight into the roles of *FvMRLKs* in the regulation of flesh fruit development and ripening.

## Materials and Methods

### Identification of MRLK proteins in *Fragaria vesca*

Annotated strawberry MRLK proteins were identified in the following three public databases: the National Center for Biotechnology Information (NCBI; http://www.ncbi.nlm.nih.gov/), the Strawberry Genome Browser (https://strawberry.plantandfood.co.nz/index.html), and PLAZA (http://bioinformatics.psb.ugent.be/plaza/). Specifically, to identify the *MRLK* gene family, we first identified the HMM profile of the malectin domain (pfam11721) and malectin-like domain (pfam12819) from the protein families database (http://pfam.janelia.org); Based on the HMM profile of the malectin and malectin-like domains, we obtained the MRLK protein candidates by searching the database of strawberry genome (https://strawberry.plantandfood.co.nz/index.html) and the MRLK protein candidates were further identified and characterized in the NCBI database for a final confirmation of the MRLK proteins. The lengths of amino acid sequences, molecular weights, and isoelectric points of the putative proteins were calculated using tools provided at the ExPasy website (http://web.expasy.org/protparam/). The signal peptides of the strawberry MRLK proteins were predicted based on the SignalP 4.1 server (http://www.cbs.dtu.dk/services/SignalP/) [34]. Information of the *Arabidopsis MRLK* gene family was obtained from The Arabidopsis Information Resource (http://www.arabidopsis.org/) and used for the comparative study.

### Phylogenetic analysis and classification of the MRLK protein family in Fragaria vesca

All identified *MRLK* genes (*FvMRLKs*) in *Fragaria vesca* were classified into different groups based on the alignment of FvMRLK proteins using ClustalX 2.1with default settings [35]. The phylogenetic trees were constructed using the neighbor-joining method implemented in MEGA 5.0[36]. Bootstrap values were calculated for 1000 iterations.

### Chromosomal location of *FvMRLKs*

All *FvMRLK* genes were mapped to strawberry chromosomes based on information available at the National Center for Biotechnology Information (NCBI; http://www.ncbi.nlm.nih.gov/). The map was drafted using Mapchart software (http://www.wageningenur.nl/en.htm) [37]. Tandem duplicated *FvMRLK* genes in the strawberry genome were identified by checking their physical locations on individual chromosomes and were defined as adjacent paralogous genes with no more than one intervening gene. Physical chromosomal locations were graphically represented to scale on the seven chromosomes.

### Analysis and distribution of conserved motifs and exon-intron structures

The exon-intron organization of the *FvMRLK* genes was determined using PLAZA (http://bioinformatics.psb.ugent.be/plaza/). The Fancy Gene online tool (http://bio.ieo.eu/fancygene/) was used to illustrate the exon-intron structure of the *FvMRLKs* [38]. The Expectation Maximization for Motif Elicitation online program (http://meme-suite.org/) [39] for DNA and protein sequence analysis was used to identify conserved motifs in the 62 FvMRLK-deduced proteins. The optimized parameters of Expectation Maximization for Motif Elicitation were set as follows: the number of repetitions, any; the maximum number of motifs, 50; and the optimum width of each motif, between 6 and 300 residues.

### Plant materials and treatments

The strawberry (*Fragaria vesca*‘Ruegen’) plants were cultivated at 22±1°C in a 13/11 h light/dark photoperiod in the Tissue Culture Center at Beijing University of Agriculture. To investigate *FvMRLK* expression in different strawberry tissues, the roots, stems, leaves, and fruits were independently sampled and analyzed using qRT-PCR. Root samples were taken from white, new roots, stem samples were taken from new creeping stems, leaf samples were the leaves 15 days old, fruit samples were taken from medium green fruits. To study the expression of *FvMRLKs* at different developmental stages, strawberry fruits were divided into five developmental stages, i.e., SG: small green stage; MG: medium green stage; LG: large green stage; TN: turn stage; and FR: full red stage, and were independently sampled. At each developmental stage, ten representative fruits were sampled, snap-frozen in liquid nitrogen, and kept at -80°C for subsequent analysis. The expression of the *FvMRLK* genes in the five fruit developmental stages was analyzed using qRT-PCR. MG fruits were selected to study some of the high-expression genes of fruit in response to various environmental cues and internal signals, including heat treatment (40°C,4 h), cold treatment (4°C,48 h), dehydration treatment (25°C,8 h), ABA treatment (25°C,100μM, 6 h), IAA treatment (25°C,500μM, 6 h),and sucrose treatment (25°C,100mM, 6 h).The fruits were cut in half with one-half used for the treatment and the other half as a control. The controls were maintained at 25°C and100% relative humidity. Three biological replicates were used in all experiments. The expression data in the heat-map were calculated as log_2_ fold change in treated vs. untreated samples.

### RNA preparation and quantitative RT-PCR analysis

Total RNA was isolated using a Plant RNA Kit (Omega) according to the manufacturer’s instructions. Quantitative RT-PCR was conducted on a LightCycler 96 Real Time PCR System (Roche Diagnostics GmbH, Mannheim City, Germany) using SYBR-Green (Takara, Dalian, China).The oligonucleotide primers were designed based on the predicted coding region using Beacon designer software and are listed in S1 Table. Primers were checked using the BLAST tool of NCBI and the dissociation curve was analyzed after the PCR reaction for primer specificity. In addition, the specificity of PCR products was verified by cloning the relative amplicons in the pMD19-T vector (Takara), sequencing, and aligning them onto the reference genome. Each reaction was carried out in a volume of 20μL, which contained 10μL SYBR-Green master mix, 8.6μL ddH_2_O, 1μL diluted template (1μL of the generated first-strand cDNA was diluted in 9 μL ddH_2_O), and 0.2μL of each gene-specific primer. The following program conditions were used: 95°C for 600 s (pre-denaturation) followed by 40 cycles at 95°C for 20 s (denaturation), 53°C for 20 s (primer annealing), and 72°C for 20 s (extension and data acquisition). Three technical replicates were performed for each biological replicate. The *actin-7* gene (LOC101313051), which maintained almost constant expression levels under all experimental conditions, was used as an internal control.

## Results

### Identification and annotation of *MRLK* genes in the strawberry genome

To identify *FvMRLK* subfamily genes, we first obtained the HMM profile of the malectin domain (pfam11721) and malectin-like domain (pfam12819) from the pfam protein families database. Using the malectin or malectin-like domain information, we searched three databases, the National Centre for Biotechnology Information, the Strawberry Genome Browser, and PLAZA. We identified a total of 100 candidate *FvMRLK* genes in the *Fragaria vesca* genome. The 100 *FvMRLK* candidates were further examined by searching the malectin or malectin-like domain in the NCBI database and 62 members were confirmed to be *FvMRLKs*, which were named *FvMRLK1* to *FvMRLK62* based on their chromosome location. The characteristics of the FvMRLK proteins are listed in Table 1 and include the deduced protein length, molecular weight, isoelectric point (PI), aliphatic index, and the grand average of hydropathicity. The deduced length of the FvMRLK proteins ranged from 132 (FvMRLK16) to 1,955 (FvMRLK 51) amino acid residues, whereas the PI ranged from 4.54 (FvMRLK33*)* to 8.88 (FvMRLK55). Thirty-one FvMRLK members contained a signal peptide (S2 Table).

**Table 1:** Strawberry MRLK genes identified in the Fragaria vesca.

Proposed name	Gene	Locus name	Chr	ORF (aa)	MW(kDa)	pI	Ai	GRAVY	strand	Group
*FvMRLK 1*	gene26561	XP_004288865	1	1016	114893.9	6.72	75.53	-0.624	+	VI
*FvMRLK 2*	gene11357	XP_004287503	1	890	98998.4	5.62	89.15	-0.188	-	III
*FvMRLK 3*	gene28050	XP_011462047	1	639	70994.6	5.98	93.79	-0.179	-	IV
*FvMRLK 4*	gene24016	XP_011463697	1	858	95229.8	5.83	86.93	-0.164	+	II
*FvMRLK 5*	gene24017	XP_004289187	1	814	90517.6	6.19	88.5	-0.153	+	II
*FvMRLK 6*	gene16334	XP_011465668	1	1187	128931.7	7.77	79.94	-0.2	+	IV
*FvMRLK 7*	gene05569	XP_004288317	1	783	87567.3	4.76	99.03	-0.122	+	III
*FvMRLK 8*	gene20704	XP_011468416	1	1086	123288.5	6.43	80.22	-0.723	-	VI
*FvMRLK 9*	gene21850	XP_004289649	2	1461	161379	7.16	77.67	-0.293	+	II
*FvMRLK10*	gene17333	XP_011458098	2	844	92261.1	5.3	96.34	0.012	+	II
*FvMRLK11*	gene17626	XP_011458187	2	868	96529.3	6.1	100.48	-0.079	-	III
*FvMRLK12*	gene11624	XP_011459850	2	848	93736.8	5.95	91.84	-0.241	+	VI
*FvMRLK13*	gene19789	XP_004293373	3	971	108344.2	8.4	89.17	-0.366	+	VI
*FvMRLK14*	gene34059	XP_011461991	3	135	15483.9	9.65	96.74	-0.248	+	IV
*FvMRLK15*	gene24842	XP_011461995	3	1789	197989.3	759	95.41	-0.015	+	IV
*FvMRLK16*	gene32988	XP_011460577	3	132	14904.1	7.82	97.42	-0.071	+	V
*FvMRLK17*	gene29734	XP_011460577	3	681	74929.4	6.57	92.35	-0.072	+	V
*FvMRLK18*	gene28878	XP_011460644	3	916	102254.3	6.04	79.6	-0.343	-	II
*FvMRLK19*	gene30033	XP_011462047	3	1007	110147.1	6.6	88.68	-0.169	+	IV
*FvMRLK20*	gene27319	XP_011460815	3	1014	111185.8	5.62	94.62	-0.101	-	IV
*FvMRLK21*	gene27321	XP_011460818	3	1011	110937.6	5.41	92.2	-0.091	+	IV
*FvMRLK22*	gene27326	XP_011460817	3	966	106406.5	6.2	91.15	-0.162	+	IV
*FvMRLK23*	gene27328	XP_004295664	3	1007	110243.5	5.65	94.14	-0.062	+	IV
*FvMRLK24*	gene27329	XP_011460821	3	931	102026.6	5.24	90.09	-0.144	+	IV
*FvMRLK25*	gene27330	XP_011460823	3	759	83739.9	5.97	87.76	-0.236	+	IV
*FvMRLK26*	gene25018	XP_011461160	3	1145	125496.7	6.12	91.15	-0.092	-	II
*FvMRLK27*	gene25031	XP_011463354	3	239	27018.6	4.66	84.9	-0.091	+	V
*FvMRLK28*	gene14004	XP_004294625	3	1169	125860.1	5.37	71.2	-0.357	-	V
*FvMRLK29*	gene14005	XP_011462143	3	577	62611.1	4.75	74.33	-0.353	-	V
*FvMRLK30*	gene14010	XP_004294627	3	832	91480.3	5.54	83.73	-0.116	+	II
*FvMRLK31*	gene28048	XP_011462168	3	493	54445.5	4.84	93.33	-0.145	-	IV
*FvMRLK32*	gene28580	XP_004296071	3	484	53260.3	5.25	85.76	-0.217	+	V
*FvMRLK33*	gene05121	XP_004296244	3	468	52082.6	4.54	73.12	-0.453	+	I
*FvMRLK34*	gene05124	XP_004296245	3	860	94587.5	6.28	81.28	-0.239	+	I
*FvMRLK35*	gene22909	XP_011463352	4	756	85062.9	5.26	86.89	-0.321	+	III
*FvMRLK36*	gene28915	XP_011460364	4	731	81731.3	6.06	100.01	0.001	-	IV
*FvMRLK37*	gene05892	XP_004298162	4	576	62901.4	5.2	91.37	-0.097	-	V
*FvMRLK38*	gene03872	XP_004298306	4	602	66979.6	5.29	86.45	-0.189	-	V
*FvMRLK39*	gene04553	XP_004298352	4	791	88436.4	7.32	83.64	-0.296	+	I
*FvMRLK40*	gene23070	XP_004298555	4	375	42757	5.02	84.19	-0.36	-	III
*FvMRLK41*	gene22902	XP_004297625	4	835	92486	5.95	86.66	-0.147	-	III
*FvMRLK42*	gene22907	XP_011463354	4	1609	181729	5.63	86.52	-0.329	+	III
*FvMRLK43*	gene22908	XP_011463354	4	791	89658.1	5.97	89.67	-0.273	+	III
*FvMRLK44*	gene26060	XP_004301135	5	1434	161363.1	6.56	82.37	-0.346	+	II
*FvMRLK45*	gene09312	XP_011465700	5	489	53813.9	5.87	86.97	-0.064	+	V
*FvMRLK46*	gene16583	XP_004304753	6	878	96629.5	5.74	76.99	-0.369	+	I
*FvMRLK47*	gene13568	XP_004302537	6	883	96718.3	6.03	75.54	-0.236	-	I
*FvMRLK48*	gene18098	XP_011466646	6	969	107689.9	5.7	88.14	-0.31	+	III
*FvMRLK49*	gene25622	XP_004305253	6	762	85854.7	5.17	83.39	-0.358	+	III
*FvMRLK50*	gene04244	XP_004306057	6	885	98830.2	6.5	78.73	-0.287	+	I
*FvMRLK51*	gene04245	XP_004306058	6	1955	216885.6	7.02	79.46	-0.254	+	I
*FvMRLK52*	gene04246	XP_004306059	6	841	93694	6.31	82.87	-0.239	+	I
*FvMRLK53*	gene04247	XP_011467877	6	833	92932.8	6.65	81.46	-0.26	+	I
*FvMRLK54*	gene04250	XP_004306062	6	827	92049.3	5.76	85.83	-0.198	-	I
*FvMRLK55*	gene19246	XP_011468989	7	458	50587.8	8.88	92.93	0.017	+	II
*FvMRLK56*	gene23363	XP_004307450	7	814	90488.8	7.22	92.16	-0.044	-	II
*FvMRLK57*	gene23366	XP_011470602	7	568	63963.8	7.52	80.44	-0.278	-	V
*FvMRLK58*	gene26704	XP_004308888	7	848	94034.1	6.38	91.8	-0.081	-	II
*FvMRLK59*	gene12571	XP_004307857	7	592	64822	4.57	87.75	-0.091	-	V
*FvMRLK60*	gene13200	XP_011470689	7	829	91709.2	6.63	88.85	-0.153	-	II
*FvMRLK61*	gene06940	XP_011457436	Un	931	104255.7	5.87	89.52	-0.229	-	III
*FvMRLK62*	gene23790	XP_004309619	Un	1664	185177.7	6.25	95.77	-0.12	-	IV

Abbreviations: Ai, aliphatic index; Chr, chromosome numbers; GRAVY, grand average of hydropathicity; MW, molecular weight; ORF, open reading frame; pI, isoelectric point; UN, unknown chromosome.

### Chromosomal distribution and exon-intron organization of the *FvMRLK* genes

**Fig 1:**
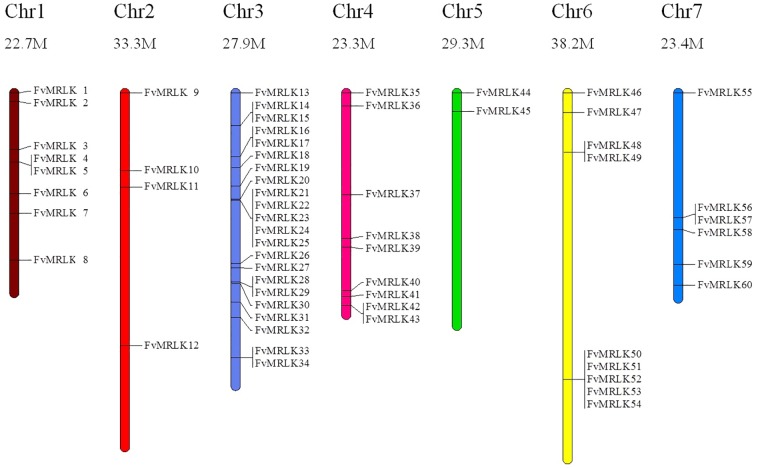
Chromosomal distribution of the FvMRLK genes. Chromosome numbers are provided at the top of each chromosome together with the approximate size. The FvMRLKs were named FvMRLK01 to FvMRLK62 based on their order on the chromosomes.

**Fig 2:**
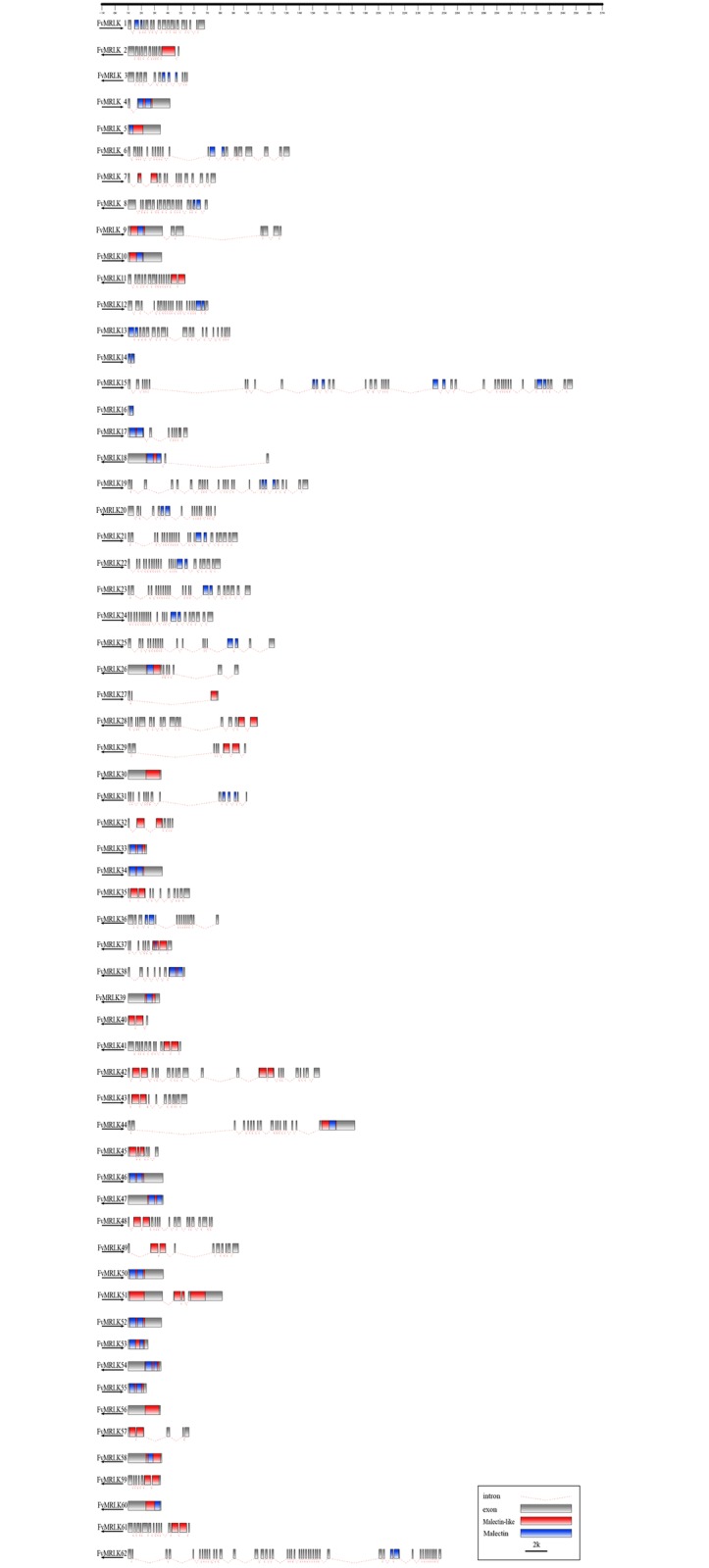
Structures and exon–intron composition of the FvMRLK genes. Names of genes are indicated on the left. Exons, represented by gray boxes, are drawn to scale. Dashed lines connecting two exons represent an intron. Malectin and malectin-like domains in the FvMRLK proteins are marked in blue and red, respectively.

The *FvMRLK* genes were mapped across the chromosomes of the strawberry genome. As shown in Fig 1, 60 of the 62 *FvMRLK* genes were distributed on chromosomes 1 through 7. Chromosome 3 (22 genes) contained the largest number of *FvMRLK* genes and chromosome 5 (2 genes) contained the smallest number of *FvMRLK* genes. We were unable to map two *FvMRLKs* (*FvMRLK61* and *FvMRLK62*) to any of the chromosomes. Based on the definition of a gene cluster by Holub [40], the *FvMRLKs* were placed into ten clusters. Among the ten clusters, five were found on chromosome 3 and two on chromosomes 6, whereas only one cluster was located on chromosomes 1, 4, and 7. Tandem and segmental duplications have been suggested as two main causes for gene family expansion in plants [41]. Five clusters of *FvMRLKs* with tandem duplications were found to be located on chromosome 3. To characterize the evolution of the *FvMRLK* family genes in strawberry, we analyzed the exon-intron structures of all *FvMRLKs*. We found that the intron numbers varied greatly (from 0 to 47) (Fig 2) amongst members of this gene family. For example, while *FvMRLK5* had no introns, *FvMRLK62* had 47. Interestingly, it appeared that the *FvMRLKs* within the same group generally had a similar exon-intron structure.

### Phylogenetic analysis, classification, and motifs of the strawberry *MRLK* gene family

**Fig 3:**
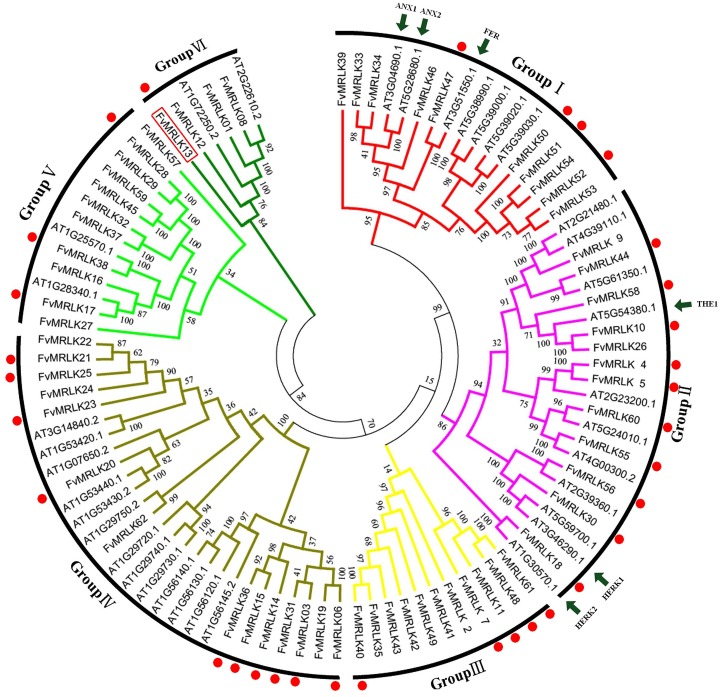
Phylogenetic tree of the MRLKs based on an alignment of strawberry and Arabidopsis protein sequences. The un-rooted phylogenetic tree was constructed with ClustalX 2.1 using the neighbour-joining method implemented in MEGA 5.0. Reliability of the predicted tree was tested using bootstrapping with 1000 replicates. Branch lines with different colors represent different MRLK groups. The functionally-elucidated homologs from Arabidopsis are labeled by arrows and the FvMRLKs expressed in fruits are labeled by red-spots.

**Fig 4:**
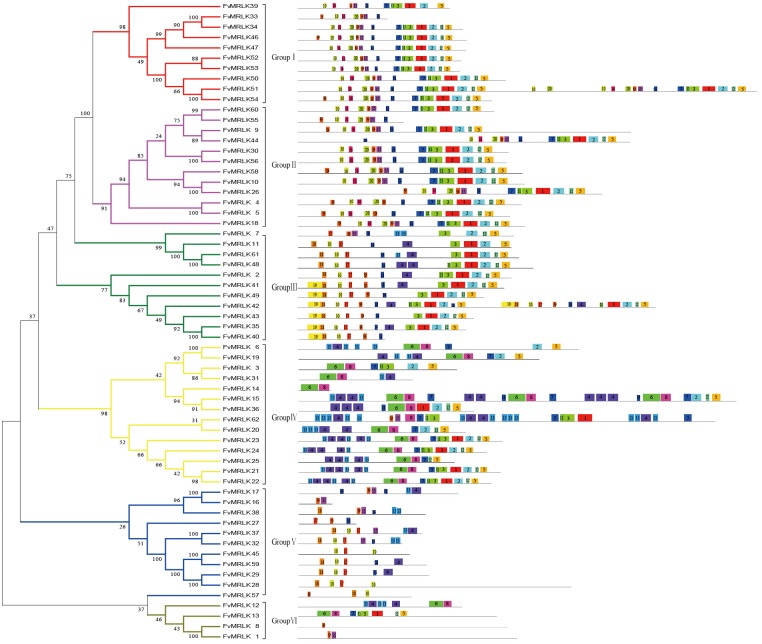
Phylogenetic tree of deduced FvMRLK proteins associated with the motif compositions in the amino-acid sequences. The phylogenetic trees were constructed using the neighbour-joining method implemented in MEGA 5.0. Reliability of the predicted tree was tested using bootstrapping with 1000 replicates. Numbers at the nodes indicate how often the group to the right appeared among bootstrap replicates. The motif composition related to each FvMRLK protein is displayed on the right-hand side. The motifs, numbered 1–20, are displayed in different colored boxes. The sequence information for each motif is provided in Table 2.

To determine the phylogenetic relationship between strawberry and *Arabidopsis MRLKs*, we constructed an unrooted phylogenetic tree based on alignments of the full-length protein sequences. The phylogenetic analyses showed that FvMRLKs could be classified into six large groups. As shown in Fig 3, groupI, II, III, IV, V, and VI contained 10, 12, 7, 14, 7, and 4 members, respectively. A major difference in the protein structure among the different groups was the number of malectin and malectin-like domains. For example, while most of the members of group I contained two malectin domains and one malectin-like domain, most of the members of group II contained one malectin domain and one malectin-like domain. Furthermore, while most of the members of group III and V contained one malectin-like domain, most of the members of group IV and VI contained only one malectin domain. Using the Multiple Expectation Maximization for Motif Elicitation online tool (http://meme-suite.org/), we identified 20 conserved motifs in the FvMRLK subfamily (Fig 4 and Table 2). Motif 6, 8, 9, 10, 14, 15, 16, 17, 18, 19, and 20 represented pivotal motifs of the malectin domains in strawberry and these conserved motifs were distributed widely amongst all FvMRLK groups. As shown in Fig 4, the FvMRLKs within the same group were found to share a similar motif composition. For example, whereas motif 19 was only found in group III, motif 10,15, and 20 were found mainly in groupI and II.

**Table 2:** Analysis and distribution of conserved motifs in FvMRLKs.

Motif	Sites	E-value	Width	Best possible match
1	38	5.1E-1440	55	L[ST]W[KE]QRL[EQ]I[CA][IV][GD]AA[RK]GL[HE]YLHTG[AC][KA]PTI[IV]HRD[VI]K[ST]TNILLDE[NK][LFW]V[AP]K[VI][SA]DFGLSK
2	42	4.8E-1120	41	TAV[KA]G[ST]FGYL[DA]PEY[YFA][RM]R[QG]QLT[ED]KSDVYSFG[VI]VL[LF]E[VI][LVI][CS][GA]RP
3	39	7.9E-947	41	HEFQ[TN]EIE[ML]LS[KR][LI][RH]HR[HN]LVSLIGYCD[ED][GK][GN][EQ][ML]ILVY[ED]YM[AE]NG
4	50	2.1E-677	41	[NS][LI][EQ]T[LI]DLS[NS]NN[LF][TS]GS[LI]PE[SF]L[GA][NK][LI][TP]TLKxL[ND][LI]SFNS[LF][ST]Gx[ILV]P
5	44	1.3E-560	34	KF[IGV]E[VIT]A[LE][KL]C[LTV]x[ED]S[GAP][VA][DE]RP[ST]M[SG]DV[LV][WS][NME]LE[GY]ALQ[LI][QE]
6	17	1.9E-560	70	[ILS]SL[TR]YY[GA][FL][CG]L[AEG]NGNYTV[KN]LHFAEI[MV][FIL][RLT][DN][ND][RT][TS]Y[KSY][SG][LV] GRR[IV]FD[VI]YIQ[GD]K[LR]VLKDF[ND]I[AR]KEAGGx[GD]K[EA][VI][IV]KEF
7	37	8.0E-572	29	F[ST][LF]A[EQ][IL][KQ]AATNNFD[EP]A[NL][KV][IL]G[EV]GGFG[PNK]VYK
8	18	1.0E-345	41	V[TSN][ENV][NS]TLEI[RH][FL][FY]WAGKGT[TC][CGN][IV]P[AR]RGTYG[PS]LISAIS[VA]T[PS]DF[KE]P
9	50	2.60E-211	15	[PS]N[FG]xYL[VI]RLHF[CA][ED][IF][EV]
10	44	1.30E-188	16	K[SD][GSA][YFV][AP]F[IV][NS][AG][IL]E[VL][VR][SP][LM]P
11	33	2.40E-165	15	TK[VI]A[VI]KR[LG][NS]PxS[EK]QG
12	38	4.60E-130	15	[CL][QH][EK]KGx[LI][ED][QE][IL][VI]DPRL
13	50	3.80E-186	21	TxLQx[LI]DLTGNYL[ST]G[PT]IPSE[IL]
14	47	5.70E-138	15	L[DP][AP][IL]LN[GA]LEI[LYF]K[IL][SN][ND]
15	29	1.10E-103	21	L[GN]Q[RL]VF[DN][VI][YF][IV]N[NG]QT[AV]ESD[AL]D[VLI]
16	23	3.80E-102	15	AL[EQ][TM][MV]YR[LVI]N[VM]GG[PS]L[IV]
17	19	1.70E-97	15	[IY]RYPDD[PV][YFH]DR[IF]W[DY][PS][FY]
18	18	1.40E-96	20	[KS]GT[KR]YL[IV]RA[ST]F[YV]YGNYDG[KQ][NGS]
19	7	7.30E-144	57	SYTEQTTGINYISD[AT]GF[IV]DTGESKLVL[PR][EY][YV][RK]D[NT]YQQPYW[SG][LV]RSFPEG[TI]RNCY[KR]INVT
20	23	9.50E-120	23	[YE][ISV]APK[EW]VYKT[AG]RSMGDN[KN]TINK[NS]

### *FvMRLK* expression profiles in strawberry

**Fig 5:**
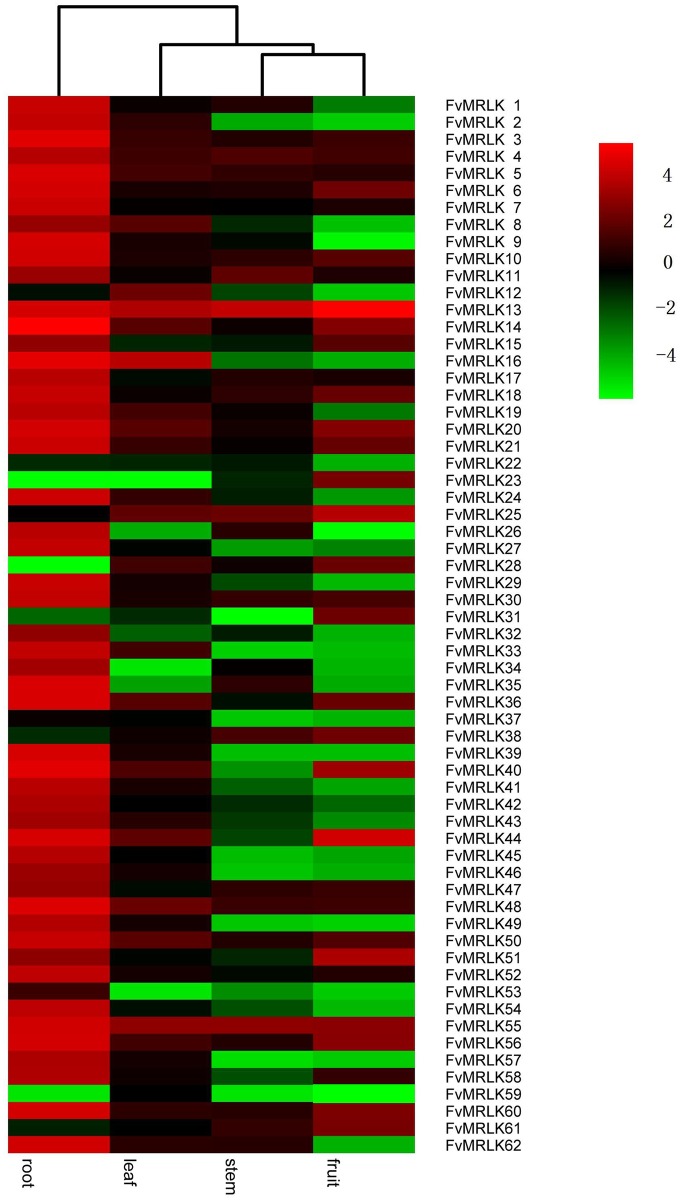
Expression profiles of the strawberry *FvMRLK* genes in different tissues. The heat map was generated based on the mean values of the qRT-PCR data from different samples. Red and green represent higher and lower expression, respectively.

**Fig 6:**
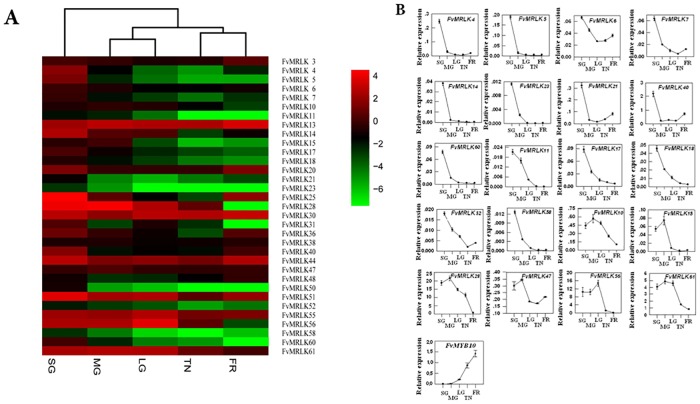
Changes in the expression profiles of *FvMRLK* genes during strawberry fruit growth and development. (a) Heat map showing the expression profiles of the *FvMRLK* genes. The heat map was generated based on the mean values of the qRT-PCR data of the different samples. Red and green colors represent higher or lower expression. (b) Expression of the *FvMRLKs* decreased during strawberry fruit growth and development. The data are the mean of 3 replicates ±SE.

The expression profiles of the *FvMRLKs* varied in different organs or tissues of strawberry. As shown in Fig 5, high levels of *FvMRLK* gene expression were found in roots. The transcript levels of more than 80% of the genes were much higher in roots than in other organs, suggesting that *FvMRLKs* is a regulator of root growth and development. Although the expression levels of most of the genes were lower in fruits than in roots, 33 members genes were found to be expressed in fruits (Fig 6a). More than 60% of these 33 genes were expressed at high levels only in the early stages of fruit development and exhibited dramatic decreases in expression as fruit development progressed (Fig 6b). While the expression of *FvMRLK*4, 5, 7, 14, 21, 23, 40 and 60 declined sharply in the earliest developmental stage (SG) [42], the expression of *FvMRLK6*, *11*,*17*,*18*, *52* and *58* dropped dramatically at the MG stage and the expression of *FvMRLK10*, *15*,*28*,*27*,*47*,*56* and *61* dropped dramatically at the LG stage. This result suggests that many *FvMRLKs* play important roles in the regulation of strawberry fruit growth and development and that there may be synergistic effects between different *FvMRLKs* that participate in these processes.

### Expression of *FvMRLKs* in response to environmental and internal cues

**Fig 7:**
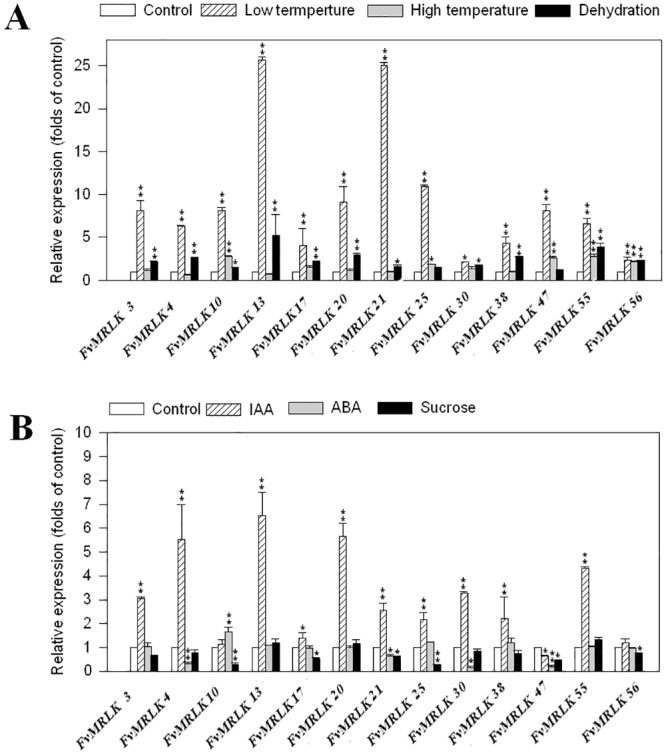
Expression of the *FvMRLKs* in response to environmental cues and internal signals that play critical roles in the regulation of strawberry fruit development and ripening. (a) Effect of environmental cues on the expression of *FvMRLKs*; (b) effect of internal signals on the expression of *FvMRLKs*. Gene expression was determined by qRT-PCR. The data are the mean of 3 replicates ±SE.

Strawberry fruit development and ripening are regulated by environmental cues, such as temperature and drought stress, and internal signals, such as auxin (IAA), abscisic acid (ABA), and sucrose. To establish whether *FvMRLKs* are indeed involved in strawberry fruit development and ripening, we examined the expression of selected *FvMRLKs* in response to IAA, ABA, and sucrose treatment as well as to temperature and dehydration stresses. As shown in Fig 7a, the expression of nearly all of the *FvMRLK* genes examined were sensitive to low temperature. The gene expression of some *FvMRLKs* increased more than 25-fold in response to low temperature treatment. Some *FvMRLKs* were also sensitive to the dehydration treatment, but the dehydration-induced changes in gene expression were generally smaller than those following low temperature treatment. As a negative regulator, IAA plays critical roles in the regulation of strawberry fruit development and ripening, whereas both ABA and sucrose have been demonstrated to be positive regulators of strawberry fruit ripening [43]. Consistent with this, IAA treatment was found to result in a dramatic increase in gene expression for most of the *FvMRLK* genes examined. By contrast, ABA and sucrose treatments resulted in a decrease in expression of some of the *FvMRLK* genes examined (Fig 7b). Collectively, these results suggest that *FvMRLKs* play important roles, and moreover, most of them function as negative regulators in the regulation of strawberry fruit development and ripening.

## Discussion

### The malectin and malectin-like domain containing RLKs family in relation to the *CrRLK1L* gene family

In *Arabidopsis*, CrRLK1Ls are a unique subfamily of RLKs comprised of 17 members. Given that CrRLK1Ls were found to contain malectin or malectin-like domains in their extracellular sequence [31], the CrRLK1Ls family has been commonly thought to be the same in terminology with the family of proteins containing malectin and malectin-like domain. In the present study, to compare FvMRLKs with the MRLKs in *Arabidopsis*, we searched the *Arabidopsis* genmone for the identification of the proteins containing the malectin and malectin-like domain. The results indicated that the *Arabidopsis* genome harbors 35 proteins containing malectin or malectin-like domains (S3 Table), which is greater than the 17 *Arabidopsis CrRLK1L* genes. Since the number of the malectin and malectin-like domain-containing proteins is much larger than that of the CrRLK1L family, it is clearly not reasonable to regard the CrRLK1L family as the family of proteins containing malectin and malectin-like domain. Because of this, in the present study, we categorized the malectin and malectin-like domain containing proteins into a unique family, and thus named as the MRLK gene family.

### Phylogenetic characteristics of *FvMRLKs*

Strawberry genome size varies by species. With 34,809 genes resulted from gene prediction modeling, the genome of diploid strawberry, *F*. *vesca*, is about twice size of the *Arabidosis* genome (240 Mb for F. vesca versus 125 Mb for *Arabidopsis*)[44]. In the present study, we identified 62 *FvMRLKs* in the strawberry genome database and 35 *MRLKs* members in the Arabidopsis genmone. Based on a phylogenetic tree generated by an alignment of the MRLK protein sequences from strawberry and *Arabidopsis*, we classified the 62 *FvMRLKs* into six major groups (Fig 3). Members within the same group shared a similar gene structure, length, and amino acid motif composition, indicating their close evolutionary relationship. *FvMRLK21*, *22*, *23*, *24*, and *25*, which were similar in motif type and the distribution and contained tandem duplications, were clustered on chromosome 3. The change in motif composition from group I to group VI may reflect the evolutionary process of the *FvMRLK* gene family. Tandem duplications and segmental/whole-genome duplications represent two of the major mechanisms for the expansion of the *RLK* gene family in plants [25].The existence of ten clustered *FvMRLK* genes indicated that gene duplications had occurred. Notably, group III was strawberry specific; no members from *Arabidopsis* were classified into this group, suggesting that this group of genes may have special roles in the regulation of growth and development in strawberry. As shown in Fig 5, among the 62 *FvMRLK* genes, 33 were found to be expressed primarily in fruits, and these fruit-expressed genes included some homologs of *FER*, *HERK1/2*, and *THE1* (i.e.,*FvMRLK10*, *FvMRLK30*, and *FvMRLK47*). Except for *FvMRLK7* and *FvMRLK40*, nine genes from group III were not expressed in the fruits, possibly implying a special role of group III genes in the regulation of vegetative growth and development in strawberry.

### Universal roles of FvMRLKs in the regulation of plant growth and development

Ripening of fleshy fruits is a complex process that involves dramatic changes in color, firmness, flavor, and aroma [43, 45]. Strawberry is a model plant for the research of the non-climacteric fruits. Limited information is available on the mechanisms controlling fruit growth and development, especially for the non-climacteric fruits. A decrease in fruit firmness is a critical event in fruit ripening. Fruit firmness is essentially controlled by cell wall metabolism and the decrease in fruit firmness primarily results from the breakdown of cellulose and pectin. The CrRLK1L protein kinases have been shown to function in cell wall metabolism [46–48]. Given the close relationship between cell wall metabolism and fruit ripening and the relationship between CrRLK1Ls and cell metabolism, CrRLK1Ls may be important regulators of fruit development and ripening. Consist with this inference, in the present study, we had found that many *MRLK* genes were specifically and highly expressed in strawberry fruits (Fig 5). Interestingly, among the *MRLKs* expressed in the fruits, the expression profiles of more than two-thirds of the *MRLKs* were found to be tightly correlated with the progress of strawberry fruit development and ripening (Fig 6). It has been well established that strawberry fruit development and ripening is regulated by both internal and environmental cues [49]. We examined the expression of *MRLKs* in response to these cues, such as IAA, ABA, sucrose, temperature and dehydration stresses. As shown in Fig 7A, most of the genes examined were sensitive to low temperature treatment, for example, in response to low temperate treatment, the expression of *FvMRLK13* and *FvMRLK21* increased to more than 25 folds compared with the control. The expression of *FvMRLKs* were also found to be strongly regulated by the internal cues. Furthermore, whereas treatments of ABA and sucrose, two positive signals controlling strawberry fruit ripening [42,49,50], caused an increase in the expression of some genes, the treatment of IAA [51], negative signal of fruit ripening, contrarily caused a dramatic increase in the gene expression (Fig 7B). Collectively, these data implies a potential role of MRLKs in the regulation of strawberry fruit development and ripening.

FER, HERK1/2, ANX1/2, and THE1 are several representative proteins that have been functionally elucidated in the *CrRLK1L* gene family [31]. FER together with its two closest homologs ANX1/2, controls polarized cell growth, thereby playing a critical role in the regulation of pollen tube and root hair elongation [52, 53]. THE1 functions to mediate the responses of plant cells to the perturbation of cellulose synthesis [24]. Acting redundantly with THE1, HERK1/2 also functions to regulate cell elongation. FER, HERK1/2, ANX1/2, and THE1 may have important roles in universal biological processes. For example, FER might be implicated in ethylene biosynthesis [16], starch accumulation[17], seed development[18],vegetative growth[21, 23], mechanical signal transduction[54], and pathogen resistance[19, 20]. Given the importance of FER, HERK1/2, ANX1/2, and THE1, identification of their homologs in the strawberry genome is important for understanding the roles of FvMRLKs in regulating strawberry plant growth and development. As shown in Fig 3, phylogenetic analysis showed that the homologs of AXN1/2, FvMRLK33, and FvMRLK34, and the homologs of FER and FvMRLK47, are in group I. Three homologs of THE1, FvMRLK10, FvMRLK26, and FvMRLK58, and one homolog of HERK1, are in group II, implying the importance of members of group I and group II in the regulation of strawberry growth and development.

Analysis of the expression profiles of the *FvMRLK* genes indicated that more than 80% of the *FvMRLKs* were expressed in roots with transcript levels much higher than in other tissues, suggesting that *FvMRLKs* play a major role in the regulation of root growth and development (Fig 5). Further analysis showed that among the 62 *FvMRLK* genes, only 33 were expressed in the early stages of fruit development. Interestingly, among these 33 genes, the transcript levels of more than 20 genes were found to be dramatically decreased at advanced stages of fruit development. Strawberry fruit ripening is sensitive to both environmental and internal cues. Low temperature inhibits ripening, whereas high temperature promotes it. IAA, ABA, and sucrose are key signals controlling strawberry fruit ripening; thus, we examined the responses of some *FvMRLKs* to these compounds. As expected, the expression of many *FvMRLKs* was found to be sensitive to the environmental cues and the internal signals. These observations suggest that *FvMRLKs* play important roles in the regulation of strawberry fruit development and ripening.

Specific expression of a gene in a tissue or cell may imply that the gene plays a specific role in that tissue or cell. To identify the *FvMRLKs* implicated in strawberry fruit development and ripening, we hoped to identify *FvMRLKs* that were only expressed in fruits but not in other tissues. However, nearly all of the *FvMRLKs* that were expressed in fruits were also found to be expressed in other organs or tissues, especially in roots, although only about half of the *FvMRLKs* were expressed in the fruits. Assuming that some *FvMRLKs* are critical regulators of fruit development and ripening, these FvMRLKs may play different roles in different tissues or cells. This was demonstrated in a recent study. For example, OST1 is a key protein controlling stomatal movement and the ortholog of OST1 also plays a critical role in the regulation of strawberry fruit ripening. Similarly, while FER was found to play a critical role in the regulation of pollen tube growth and sperm release, it also plays a critical role in the regulation of root hair elongation as well as many other biological processes as described above. Based on the expression profile of the *FvMRLK* genes, further investigation is needed to establish how different *FvMRLKs* coordinately regulate a common biological process, and by contrast, how individual *FvMRLK* genes regulate distinct biological processes. Collectively, the present study provides valuable information about *FvMRLKs* in strawberry, and thereby casts light on the mechanisms underlying the regulation of fruit development and ripening in this model non-climacteric fruit.

## Conclusions

Sixty-two *FvMRLKs* were characterized in the strawberry genome, and classified these genes into six subfamilies with distinct malectin domains in the extracellular regions of the encoded proteins. More than 80% of the *FvMRLKs* were expressed in various tissues, with higher levels in roots than in other organs. Thirty-three *FvMRLKs* were expressed in fruits during the early stages of development, and over 60% of these exhibited dramatic decreases in expression during fruit growth and development. Moreover, the expression of some *FvMRLKs* was sensitive to both environmental and internal cues that play critical roles in regulating strawberry fruit development and ripening.

## Supporting Information

S1 Table Primer sequences used for qRT-PCR analyses. (DOCX)

S2 Table List of 31 FvMRLK proteins containing a signal peptide. (DOCX)

S3 Table *MRLK* genes identified in Arabidopsis thaliana. (DOCX)

## Abbreviations

SG: small green stage
MG: medium green stage
LG: large green stage
TN: turn stage
FR: full red stage
