# Supplementary material for: Genome-Wide Identification and Expression Analysis of MRLK Family Genes Associated with Strawberry (Fragaria vesca) Fruit Ripening and Abiotic Stress Responses
Source: PLoS One. 2016 Sep 29;11(9):e0163647. doi: 10.1371/journal.pone.0163647 (PMC5042409; doi:10.1371/journal.pone.0163647)
Supplement: S1 Table — (DOCX) [file pone.0163647.s001.docx]

S1 Table: List of thirty-one FvMRLK proteins containing signal peptide

| **sequence-name** | **source** | **feature** | **start** | **end** | **score** |
| --- | --- | --- | --- | --- | --- |
| FvMRLK 2 | SignalP-4.1 | SIGNAL | 1 | 22 | 0.915 |
| FvMRLK 5 | SignalP-4.1 | SIGNAL | 1 | 30 | 0.641 |
| FvMRLK 6 | SignalP-4.1 | SIGNAL | 1 | 22 | 0.889 |
| FvMRLK 7 | SignalP-4.1 | SIGNAL | 1 | 29 | 0.889 |
| FvMRLK10 | SignalP-4. | SIGNAL | 1 | 26 | 0.839 |
| FvMRLK21 | SignalP-4.1 | SIGNAL | 1 | 26 | 0.855 |
| FvMRLK23 | SignalP-4.1 | SIGNAL | 1 | 27 | 0.919 |
| FvMRLK30 | SignalP-4.1 | SIGNAL | 1 | 22 | 0.770 |
| FvMRLK32 | SignalP-4.1 | SIGNAL | 1 | 19 | 0.904 |
| FvMRLK33 | SignalP-4.1 | SIGNAL | 1 | 24 | 0.850 |
| FvMRLK34 | SignalP-4.1 | SIGNAL | 1 | 24 | 0.862 |
| FvMRLK35 | SignalP-4.1 | SIGNAL | 1 | 24 | 0.840 |
| FvMRLK38 | SignalP-4.1 | SIGNAL | 1 | 19 | 0.917 |
| FvMRLK40 | SignalP-4.1 | SIGNAL | 1 | 25 | 0.916 |
| FvMRLK41 | SignalP-4.1 | SIGNAL | 1 | 22 | 0.898 |
| FvMRLK42 | SignalP-4.1 | SIGNAL | 1 | 27 | 0.788 |
| FvMRLK43 | SignalP-4.1 | SIGNAL | 1 | 30 | 0.686 |
| FvMRLK45 | SignalP-4.1 | SIGNAL | 1 | 27 | 0.786 |
| FvMRLK46 | SignalP-4.1 | SIGNAL | 1 | 16 | 0.722 |
| FvMRLK47 | SignalP-4.1 | SIGNAL | 1 | 19 | 0.733 |
| FvMRLK48 | SignalP-4.1 | SIGNAL | 1 | 19 | 0.918 |
| FvMRLK50 | SignalP-4.1 | SIGNAL | 1 | 23 | 0.765 |
| FvMRLK51 | SignalP-4.1 | SIGNAL | 1 | 23 | 0.824 |
| FvMRLK52 | SignalP-4.1 | SIGNAL | 1 | 23 | 0.849 |
| FvMRLK53 | SignalP-4.1 | SIGNAL | 1 | 22 | 0.784 |
| FvMRLK54 | SignalP-4.1 | SIGNAL | 1 | 22 | 0.798 |
| FvMRLK55 | SignalP-4.1 | SIGNAL | 1 | 23 | 0.821 |
| FvMRLK56 | SignalP-4.1 | SIGNAL | 1 | 25 | 0.878 |
| FvMRLK58 | SignalP-4.1 | SIGNAL | 1 | 24 | 0.756 |
| FvMRLK60 | SignalP-4.1 | SIGNAL | 1 | 20 | 0.821 |
| FvMRLK61 | SignalP-4.1 | SIGNAL | 1 | 20 | 0.874 |
