# Supplementary material for: Genome-Wide Identification and Expression Analysis of MRLK Family Genes Associated with Strawberry (Fragaria vesca) Fruit Ripening and Abiotic Stress Responses
Source: PLoS One. 2016 Sep 29;11(9):e0163647. doi: 10.1371/journal.pone.0163647 (PMC5042409; doi:10.1371/journal.pone.0163647)
Supplement: S2 Table — (DOCX) [file pone.0163647.s002.docx]

S2 Table: MRLK genes identified in the [Arabidopsis thaliana](http://www.arabidopsis.org/portals/education/aboutarabidopsis.jsp)

| **Number** | **Gene name** | **Other name** | **Chr** | **ORF (aa)** | **MW(kDa)** | **pI** |
| --- | --- | --- | --- | --- | --- | --- |
| *1* | [AT1G07650.2](http://www.arabidopsis.org/servlets/TairObject?type=aa_sequence&id=6530307382) |  | 1 | 1020 | 113497.3 | 8.8892 |
| *2* | *AT1G25570.1* |  | 1 | 628 | 68971.6 | 5.1033 |
| *3* | *AT1G28340.1* |  | 1 | 626 | 68418.1 | 8.4523 |
| *4* | *AT1G29720.1* |  | 1 | 1019 | 112761.4 | 7.3086 |
| *5* | *AT1G29730.1* |  | 1 | 969 | 107674.3 | 7.4699 |
| *6* | *AT1G29740.1* |  | 1 | 1078 | 120051.9 | 6.936 |
| *7* | *AT1G29750.2* | RKF1 | 1 | 1021 | 113462.3 | 7.9908 |
| *8* | *AT1G30570.1* | HERK2 | 1 | 849 | 93945.9 | 6.5889 |
| *9* | *AT1G53420.1* |  | 1 | 953 | 107489.4 | 6.873 |
| *10* | *AT1G53430.2* |  | 1 | 1030 | 113975.6 | 5.314 |
| *11* | *AT1G53440.1* |  | 1 | 1035 | 114850.1 | 6.9888 |
| *12* | *AT1G56120.1* |  | 1 | 1047 | 114764.6 | 6.8876 |
| *13* | *AT1G56130.1* |  | 1 | 1032 | 113040.7 | 6.8959 |
| *14* | *AT1G56140.1* |  | 1 | 1033 | 113753.3 | 6.7811 |
| *15* | *AT1G56145.2* |  | 1 | 1039 | 114872.5 | 8.4192 |
| *16* | *AT1G72250.2* |  | 1 | 1203 | 134253.1 | 6.7904 |
| 17 | AT2G21480.1 |  | 2 | 871 | 95935.2 | 5.8549 |
| 18 | AT2G22610.2 |  | 2 | 1083 | 122921.4 | 6.2553 |
| 19 | *AT2G23200.1* |  | 2 | 834 | 93378.5 | 6.3802 |
| *20* | *AT2G39360.1* |  | 2 | 815 | 91320.6 | 6.5214 |
| *21* | *AT3G04690.1* | ANXUR1 | 3 | 850 | 94047.6 | 6.9057 |
| *22* | *AT3G14840.2* | LIK1 | 3 | 1020 | 112275.5 | 6.0779 |
| *23* | *AT3G51550.1* | FERONIA | 3 | 985 | 98148.9 | 6.1149 |
| *24* | *AT4G00300.2* |  | 4 | 785 | 88860.2 | 88860.2 |
| *25* | *AT4G39110.1* |  | 4 | 878 | 96504.0 | 6.01 |
| *26* | *AT5G28680.1* | ANXUR2 | 5 | 858 | 94295.8 | 6.9951 |
| *27* | *AT5G38990.1* |  | 5 | 880 | 97952.3 | 5.4954 |
| *28* | *AT5G39000.1* |  | 5 | 873 | 97163.5 | 5.9896 |
| *29* | *AT5G39020.1* |  | 5 | 813 | 90446.9 | 6.9361 |
| *30* | *AT5G39030.1* |  | 5 | 806 | 90670.1 | 5.7291 |
| *31* | *AT5G54380.1* | THESEUS1 | 5 | 855 | 93294.0 | 5.9623 |
| *32* | *AT5G59700.1* |  | 5 | 829 | 91963.1 | 6.0062 |
| *33* | *AT5G61350.1* | ERULUS | 5 | 842 | 92686.2 | 6.3205 |
| *34* | *AT3G46290.1* | HERK1 | 5 | 830 | 91466.8 | 6.2517 |
| *35* | *AT5G24010.1* |  | 5 | 824 | 91822.4 | 7.7507 |
|  |  |  |  |  |  |  |

Abbreviations: Chr, chromosome numbers; MW, molecular weight; ORF, open reading frame; pI, isoelectric point;
