# Supplementary material for: Genome-Wide Identification and Expression Analysis of MRLK Family Genes Associated with Strawberry (Fragaria vesca) Fruit Ripening and Abiotic Stress Responses
Source: PLoS One. 2016 Sep 29;11(9):e0163647. doi: 10.1371/journal.pone.0163647 (PMC5042409; doi:10.1371/journal.pone.0163647)
Supplement: S3 Table — (DOCX) [file pone.0163647.s003.docx]

S3 Table: Primer sequences used for real-time RT-PCR analyses.

| **Forward primer** | **Sequence** | **Reverse primer** | **Sequence** |
| --- | --- | --- | --- |
| FvActin1-F | GCG ACA ATG GAA CTG GAA TGG | FvActin1-R | GACAATTTCCCGTTCAGCAGTG |
| FvActin2-F | TGGGTTTGCTGGAGATGAT | FvActin2-R | CAGTTAGGAGAACTGGGTGC |
| FvMRLK 1-F | GCCTGCTACATTGACTGA | FvMRLK 1-R | CTTGACTGATGGAACCTGAT |
| FvMRLK 2-F | ACATAACATACTCACCAGATAACA | FvMRLK 2-R | TCACCACAGACCAGAAGT |
| FvMRLK 3-F | CTATGGTGTTGTCATTCTA | FvMRLK 3-R | TAATTCGTCTTGCTTCTT |
| FvMRLK 4-F | TTGATGTTCTGGATTCTAC | FvMRLK 4-R | GGCTATTATTACTGGTGAA |
| FvMRLK 5-F | GGTGAGACGGTTGAGAAG | FvMRLK 5-R | CAAGTGAATGAATACGCTGAA |
| FvMRLK 6-F | ATGGAACACAACAGGAGAC | FvMRLK 6-R | TGGAATTACACCGACAACTT |
| FvMRLK 7-F | GGTAGTGGAGGTAGCATTG | FvMRLK 7-R | AGTAGAAGGTGGCGGTAT |
| FvMRLK 8-F | ATGAAGGTTGAAGAGTTGGA | FvMRLK 8-R | TTATGCTGAAGAGTGGTAGAA |
| FvMRLK 9-F | CTACTCATCGGAACCATCAA | FvMRLK 9-R | TAGAAGCAGCAGCATCAG |
| FvMRLK10-F | GTCTATGCCACTGCTGAA | FvMRLK10-R | TGAATCTGCTGAGGAGTTG |
| FvMRLK11-F | TTGCTGTTGATGCTGCTA | FvMRLK11-R | GCTGTTGTGTTGAGTAGTATTC |
| FvMRLK12-F | TAGACATCTTGAGAATTGGTGAT | FvMRLK12-R | TTGGACTTACGATACTTGTAGG |
| FvMRLK13-F | GTCAAGCAAGAGCATCCA | FvMRLK13-R | TTCCAAGGCAGCAATCTC |
| FvMRLK14-F | CACGCTTTAGTCCTCTTTCTCT | FvMRLK14-R | ATCAACACCTTCTGCTTCCTT |
| FvMRLK15-F | GGTGGTGATGCTGGTTAT | FvMRLK15-R | AGGTGAGAGTGTTGTTGAAT |
| FvMRLK16-F | GTCCAATCACTGCCTCTG | FvMRLK16-R | CTTGTTAGCATCAGCATCCA |
| FvMRLK17-F | TCCATTGAAGCCAGCATTA | FvMRLK17-R | TTGTCCTACACCATTGATACTT |
| FvMRLK18-F | AAGAGAAGATGATGTTGT | FvMRLK18-R | GAGTGATGTTGTTGTTAG |
| FvMRLK19-F | TGCGTCTGCTCTGATAATGTC | FvMRLK19-R | AGTGAGTTCTCCGAGTTCCT |
| FvMRLK20-F | ACTCCGATTCAAGCACCAT | FvMRLK20-R | AGCGAGAGAAACCGAAGAA |
| FvMRLK21-F | CCAACAAGGCACAATATCTG | FvMRLK21-R | ATAACCACGCATCGCATA |
| FvMRLK22-F | CTTCTCCAATCTTATTAGC | FvMRLK22-R | GCAATCTTCATTATACTACT |
| FvMRLK23-F | GCAGCGGAGAATATGGAT | FvMRLK23-R | CAGATAGTTACGAGCAAGGT |
| FvMRLK24-F | TGTGTCTTCAGCAGTTAGG | FvMRLK24-R | TCACCATTCTGTTCTTCCAA |
| FvMRLK25-F | TGACAGAGTTGGAGACATTAGAC | FvMRLK25-R | AGTTCTTGCCGCCATTCT |
| FvMRLK26-F | CAACTCCTCAGCAGATTCA | FvMRLK26-R | GCCAAGCAGCAATACAAG |
| FvMRLK27-F | GATTGCTGCGGATGTAGG | FvMRLK27-R | TCTCATAGGCGTTCAGGAT |
| FvMRLK28-F | GCAAGCAGAGTTCAGCATAC | FvMRLK28-R | GGATTCACCAGAGGCACAT |
| FvMRLK29-F | AATGTCTTGTGCGTGGAA | FvMRLK29-R | ACTGGTTCGGCTTAGATTG |
| FvMRLK30-F | CGATGACAAGAGTGAGATGA | FvMRLK30-R | CAGTGTGAAGGTAGTGAAGT |
| FvMRLK31-F | CTGTTGGTGCTGCTCTTCT | FvMRLK31-R | TGGTGTCATCATCTTCATCTATCG |
| FvMRLK32-F | TGCCTCCTACTCCTATGG | FvMRLK32-R | TTGTTCTTGATGTTGCTTGTT |
| FvMRLK33-F | GATGCGTTGCTTAATGGATT | FvMRLK33-R | TCAGTTATCGTCAGTGTCTTG |
| FvMRLK34-F | CACCAAGGTCAATCAGAGA | FvMRLK34-R | AGTTGCCACGAGTATCATT |
| FvMRLK35-F | ACTGGAGGCTTGACATTG | FvMRLK35-R | TGCTGGTTCGTAGAGATTG |
| FvMRLK36-F | AAGGAAGAGGCAATGAGAATGAT | FvMRLK36-R | GGCGGAAGTCTCTGTTGAATA |
| FvMRLK37-F | TGTGAGCAGTTGAAGAAGAT | FvMRLK37-R | GGAGAGGAGGAGGAAGAG |
| FvMRLK38-F | CGTGCCATCTCAATAAGAATG | FvMRLK38-R | TCCAATAAGTTGTCGTTCAGTA |
| FvMRLK39-F | TCACGCCATCATCAACTC | FvMRLK39-R | TGTCTGTATCAACCTCTTCATT |
| FvMRLK40-F | ACTGGAGGCTTGACATTGG | FvMRLK40-R | TGCTGGTTCGTAGAGATTGTG |
| FvMRLK41-F | CCACAGTAGATTCCATAGTTCA | FvMRLK41-R | GGCACAAGAGGTCCATAA |
| FvMRLK42-F | GCTATCAACAACCTTACTTATCTT | FvMRLK42-R | GCATCCTTCAACCTCACT |
| FvMRLK43-F | AATACTTCATCCACAATC | FvMRLK43-R | GGTCATTATACATCTTCC |
| FvMRLK44-F | TTCAATGTCACTCTTAATG | FvMRLK44-R | ATTATCCTCCTCACCATA |
| FvMRLK45-F | CTAACCACCAGAGGACTTC | FvMRLK45-R | AGGAATGGAGGAATCATCAG |
| FvMRLK46-F | ATCATCCAAGAGTAACGGTAG | FvMRLK46-R | TCACAGAATCCAATAAGAGACA |
| FvMRLK47-F | GCTTAGGTCTTGGTATGATGA | FvMRLK47-R | TAGTTGAGGTTGATGTTGTTATTC |
| FvMRLK48-F | ATTCCATTCATTCTTCGTTA | FvMRLK48-R | TGTCATCCACAACTTCTA |
| FvMRLK49-F | CCTGAGTCTGATGATGATACGA | FvMRLK49-R | TGTGAGAGGCTTGGAGTTG |
| FvMRLK50-F | TTGCCGTGAGTTGTATGAA | FvMRLK50-R | TTAGTCGTTCTGCTGTTCTT |
| FvMRLK51-F | GCCATCTTGAACGGACTA | FvMRLK51-R | CTACACCTGCGATAATACCA |
| FvMRLK52-F | AATCCAAGCATTCAACTCAAG | FvMRLK52-R | ACCACTCCATCCGACTAT |
| FvMRLK53-F | CGAAGAAGAAGCCGACATA | FvMRLK53-R | GCAATTACACCAGCAACAA |
| FvMRLK54-F | AGCACAGAACGACACAAT | FvMRLK54-R | TCCACCACTCCAGAGAAT |
| FvMRLK55-F | AGTATGCCGTATGCTGTTGAT | FvMRLK55-R | ATCCTCCTCCGTGTTACCA |
| FvMRLK56-F | ATAGAAGAAGAAGAAGAAGAGGAA | FvMRLK56-R | ACTTGAATTGCTGCTAATGG |
| FvMRLK57-F | CGGTGGAGATGAAGTAAGG | FvMRLK57-R | GATTGTGGAGTCGGTGTT |
| FvMRLK58-F | CATTAGAGCCTGGTATAGAGATT | FvMRLK58-R | CTTGATTGAGCGTGGATTG |
| FvMRLK59-F | GCACGATAACGGCTTAGA | FvMRLK59-R | GCTGGAGGAGTAACTTGAG |
| FvMRLK60-F | GTCGGCTAACTACACCATT | FvMRLK60-R | ACTCACCAACTTAACACCAT |
| FvMRLK61-F | TTCTCAATATCCTTCGTCTCAT | FvMRLK61-R | CCATCCAACCACAACTCT |
| FvMRLK62-F | GCTATTGCTGGTCTATGAGT | FvMRLK62-R | GGTGCTTATGTGAGTCTTCT |
